# Redefining the immune landscape of hepatitis A virus infection

**DOI:** 10.1038/s12276-025-01431-2

**Published:** 2025-04-02

**Authors:** Ombretta Colasanti, Hosun Yu, Volker Lohmann, Eui-Cheol Shin

**Affiliations:** 1 Heidelberg University, Medical Faculty Heidelberg, Department of Infectious Diseases, Molecular Virology, Section Virus–Host-Interactions, Center for Integrative Infectious Disease Research, Heidelberg, Germany; 2 Graduate School of Medical Science and Engineering, Korea Advanced Institute of Science and Technology, Daejeon, Republic of Korea; 3 The Center for Viral Immunology, Korea Virus Research Institute, Institute for Basic Science, Daejeon, Republic of Korea

**Keywords:** Viral infection, Infection

## Abstract

Despite the development of effective vaccines against hepatitis A virus (HAV) infection, outbreaks of acute hepatitis A still occur globally, such that HAV remains a major cause of acute viral hepatitis. Most patients with acute hepatitis A recover spontaneously; however, some adult cases result in acute liver failure due to immune-mediated liver damage. Previous studies suggested that HAV evades the innate immune response through strong counteractive mechanisms, and that HAV-specific CD8^+^ T cells contribute to liver damage in patients with acute hepatitis A. However, recent research findings have led to revisions of old hypotheses. Here we will describe the most current knowledge regarding the innate immune response to HAV and the HAV-mediated counteractions against innate immune responses. Additionally, we will discuss the roles of various types of T cells in viral clearance and liver injury in patients with acute hepatitis A.

## Redefining the immune landscape of hepatitis A virus infection

Hepatitis A virus (HAV) is a small virus that causes liver infections, often spread through contaminated food and water. Despite effective vaccines, understanding how HAV interacts with the immune system is crucial. This study focuses on how HAV triggers the immune system and how this can lead to liver damage. The authors used cell cultures and animal models to study the effects of HAV and found that HAV can activate certain immune cells, such as CD8^+^ T cells, which can attack liver cells, causing damage. This activation is partly due to IL-15, which boosts immune cell activity. The results show that while the immune response helps clear the virus, it can also harm the liver. The researchers conclude that balancing this response is key to managing HAV infections. This summary was initially drafted using artificial intelligence, then revised and fact-checked by the author.

## Introduction

**Fig. 1:**
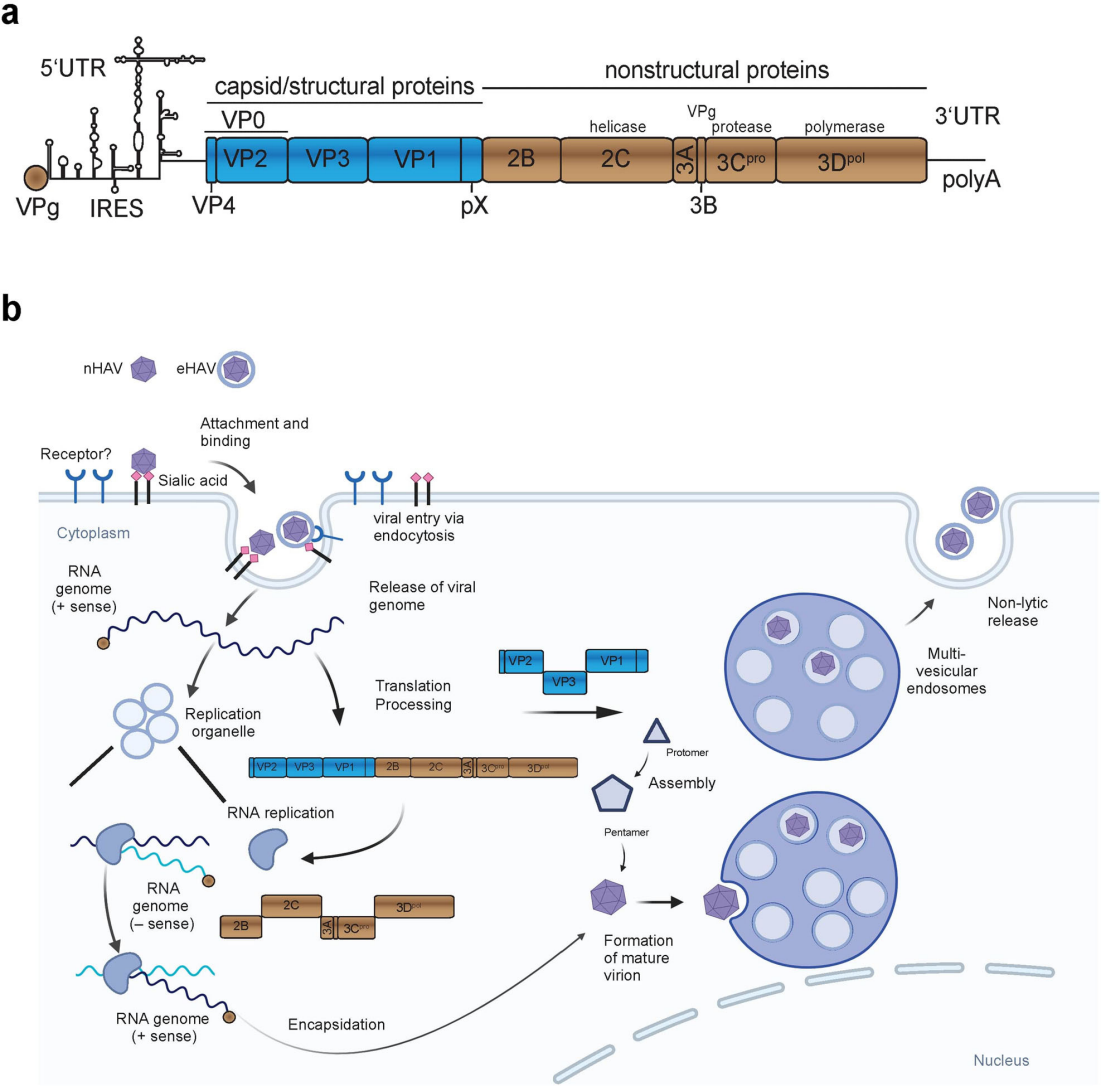
HAV genome organization and replication cycle. **a** A schematic representation of the HAV genome. The single-stranded positive-sense RNA genome is covalently linked to a viral protein (3B, VPg), and encodes a single polyprotein, flanked by UTRs. The 5′ UTR contains an IRES that facilitates translation. The N-terminus of the polyprotein encodes the capsid proteins VP1–VP4. In mature nHAV virions, the pX moiety of VP1 is cleaved off by an unknown host protease. VP0 is cleaved after packaging of the HAV genome. The C-terminal portion of the polyprotein encodes nonstructural proteins required for RNA replication. **b** HAV replication cycle. eHAV and nHAV virions are depicted on top. A bona fide entry receptor for nHAV or eHAV has not yet been identified, but recent studies suggest that sialic acid and gangliosides play important roles in viral entry^9,10^. The VPg-linked genome serves as mRNA, and translation is mediated by the IRES. Polyprotein processing is mainly mediated by the viral protease 3C, except for the autocatalytic VP4/VP2 cleavage, and involves multiple precursor proteins with partially distinct functions. The nonstructural proteins are engaged in viral RNA replication, which is associated with vesicular–tubular membrane alterations. Analogous to other picornaviruses, both positive- and negative-strand RNA synthesis is supposed to be primed with VPg/3B, which is tethered to mitochondrial membranes by 3A. Newly synthesized positive-strand genomes are packaged into virions, and eHAV is enveloped and noncytopathically secreted via multivesicular endosomes. Note that the pseudo-envelope is shed off by bile salts in bile canaliculi, such that eHAV can be secreted across the apical membrane and then release nHAV (not shown).

Hepatitis A virus (HAV), first identified in 1973^1^, is a small non-enveloped virus in the *Picornaviridae* family, with a positive-strand RNA genome of 7,500 nucleotides in length^2,3^. HAV exhibits high antigenic conservation, with only one serotype and seven genotypes, of which four are known to infect humans^4^. The genome is organized into a single open-reading frame flanked by untranslated regions (UTRs)^5,6^, and encodes structural proteins that form the icosahedral capsid, and nonstructural proteins involved in replication and host interaction. The 5′ UTR contains a type-III internal ribosome entry site (IRES), which facilitates cap-independent translation, while the 3′ UTR is terminated by a poly(A) tail (reviewed in refs. ^7,8^) (Fig. 1a). The structural proteins VP1, VP2, VP3 and VP4 form the capsid, while the nonstructural proteins 2B, 2C, 3A, 3B, 3C and 3D are involved in viral replication, polyprotein processing and host interactions (reviewed in ref. ^7^).

HAV primarily replicates in hepatocytes after entering cells via clathrin-dependent endocytosis^9,10^. In the cytoplasm of the host cell, the positive-sense RNA genome acts as mRNA and is translated into a polyprotein, which is cleaved into individual proteins by the virus-encoded 3C proteinase (3C^pro^). As with other positive-strand RNA viruses, HAV RNA replication is associated with virus-induced membranous replication organelles, and involves a double-stranded (ds) RNA intermediate. HAV exists in two forms: naked HAV (nHAV) and quasi-enveloped HAV (eHAV)^11^ (Fig. 1b). All virions are released as eHAV through a noncytolytic process, comparable to exosome secretion (recently reviewed in ref. ^9^), and then the quasi-envelope is stripped from eHAV by bile acids in the proximal bile canaliculi. The nHAV form is highly stable and able to persist in the environment, which facilitates fecal–oral transmission (reviewed in refs. ^8,9^).

Clinically, HAV infection is highly contagious and typically self-limiting. Symptoms include fever, malaise, vomiting, nausea and jaundice (reviewed in ref. ^12^). Fulminant hepatitis requiring liver transplantation occurs in less than 1% of cases^13^. Although acute hepatitis A (AHA) does not become chronic, severe cases can lead to liver failure, especially among adults and patients with underlying conditions^13^. Globally, HAV causes over 100 million infections and 15,000–30,000 deaths every year, with transmission primarily occurring via contaminated food and water^14^. HAV-infected individuals produce virus-neutralizing HAV-specific antibodies that provide lifelong protective immunity to those who recover^15^. These neutralizing antibodies are also generated following immunization with inactivated virus-based vaccines. Vaccination has been available since the 1990s, and has notably controlled HAV infection rates, with inactivated and live attenuated vaccines yielding high efficacy and long-lasting immunity^16^.

Despite the effectiveness of vaccines, it remains critical to understand the interactions between HAV and the host’s immune system. Historically, HAV was thought to strongly abrogate the innate immune response in infected cells^17–24^. However, recent studies have revealed that HAV mounts a robust cell-intrinsic innate immune response in infected hepatocytes^25^. Moreover, recent studies demonstrate that HAV infection causes bystander CD8^+^ T cell activation, and aberrant changes in the regulatory T (Treg) cell population^26,27^. The present review summarizes the current knowledge of the immune response (particularly innate and T cell responses) to HAV, in the light of recent research findings, providing an updated perspective and identifying gaps for future research.

## HAV-induced innate immune response in cell culture system in vitro

The typical clearance of HAV infections, together with the HAV vaccine efficacy, suggest that HAV induces a robust immune response; however, this has only been investigated to a limited extent in vitro and in vivo. In contrast to the outcome of in vivo acute infection, in vitro HAV culturing is typically marked by slow and persistent infection owing to the long adaptation required for HAV to grow in culture and the noncytopathic phenotype^28^. Following the adaptation of HAV to cell culture, several cell lines have been proven capable of supporting HAV infection—including marmoset primary hepatocytes, the human hepatoma cell line Alexander (PLC/PRF/5) and fetal rhesus kidney cells (FhrK6 and FhrK4). However, studies in these infection models have focused on detecting HAV replication, potential cytopathic effects or the impacts of specific mutations in the viral genome, neglecting the innate immune response (reviewed in ref. ^29^). After the publication of contradictory findings regarding interferon (IFN) responses in patients with HAV infection (reviewed in ref. ^29^), in 1985, researchers measured type I IFNs α and β in HAV-infected human embryo fibroblasts. No IFN secretion was detected, hinting that HAV could potentially hinder the innate immune response. This possibility was further supported by the finding that HAV-infected fibroblasts could not abrogate infections by other viruses (reviewed in ref. ^30^), and the observation that even small amounts of exogenous IFN-α/β could eradicate persistent HAV infections in human fibroblasts (reviewed in ref. ^30^). Together, this evidence prompted further investigations of this lack of induction.

**Fig. 2:**
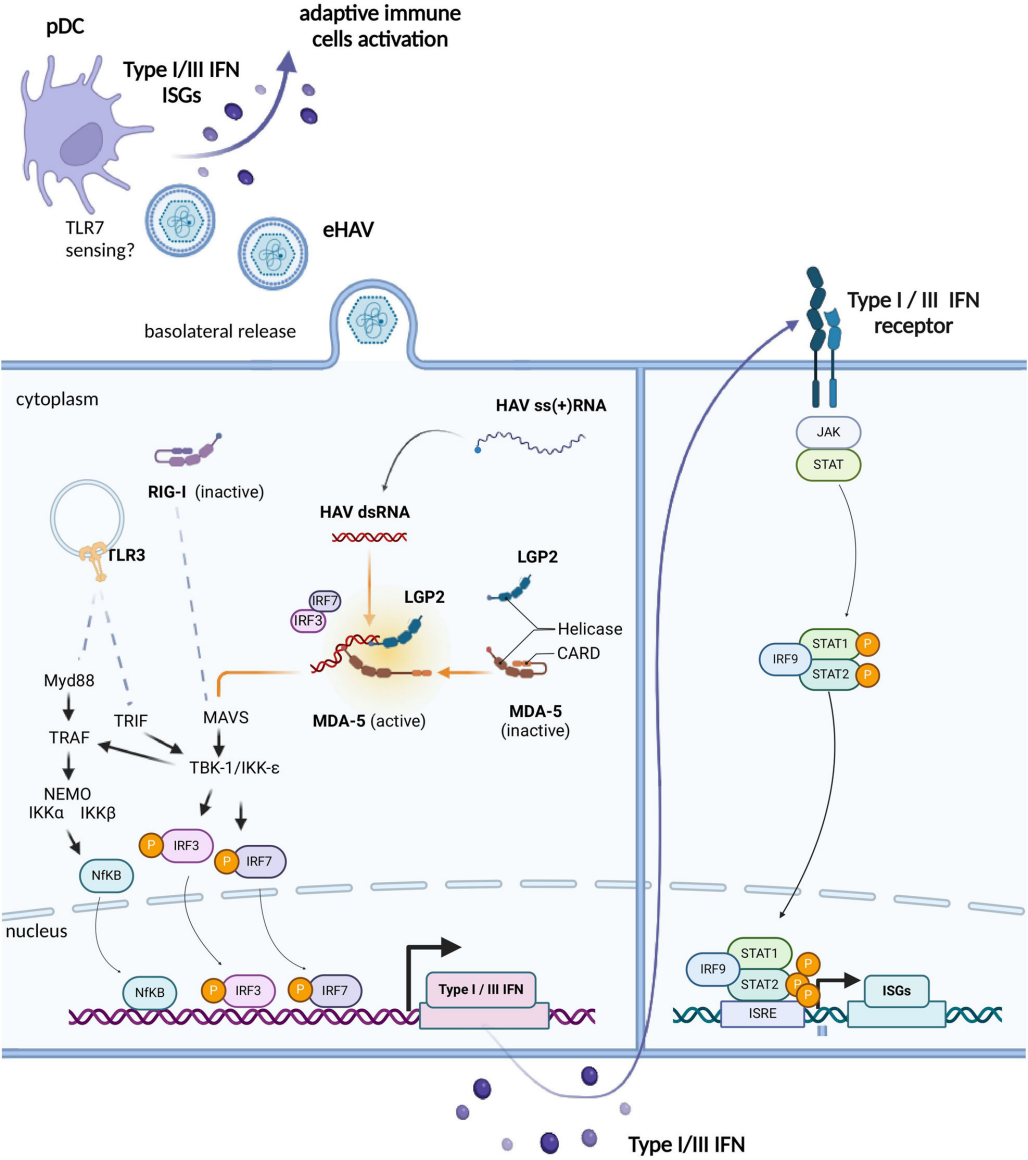
Innate immune induction by HAV in vitro. A schematic representation of the simplified key signaling pathways involved in HAV sensing. Top: pDCs can sense the eHAV particles that basolaterally egress from infected hepatocytes, through an unknown mechanism (hypothetically involving TLR7), resulting in the induction of ISGs and IFN. Middle: the current literature indicates that the dsRNA intermediate of HAV replication is primarily detected by MDA5. LGP2 enhances the stability of MDA5 binding to HAV dsRNA. Upon sensing HAV dsRNA, MDA5 undergoes conformational changes that expose its N-terminal CARD. The exposed CARD domains then interact with the MAVS adapter molecule on the mitochondria, triggering a downstream signaling cascade. MAVS recruits and activates TBK1 and IKKε, which then phosphorylate transcription factors, including IRF3, IRF7 and NFκB. These phosphorylated transcription factors translocate to the nucleus, where they promote transcription of genes encoding IFNs and ISGs. The downstream signaling involves secreted IFNs binding to the cell-surface IFNAR receptor, thereby activating the JAK–STAT pathway. This leads to STAT1 and STAT2 phosphorylation, which then form the ISGF3 complex with IRF9. The ISGF3 complex translocates to the nucleus, and binds IFN-stimulated response elements (ISREs) in the promoters of ISGs, driving their expression. RIG-I has not been shown to be involved in HAV dsRNA detection, and is thus schematized in its inactive form. TLR3 can senses potential dsRNA within the endosomal lumen, but this has not been demonstrated for HAV. Upon recognition, TLR3 recruits the adapter protein TRIF, which activates TBK1 and IKKε, similar to the RLR pathway. TRIF also activates NEMO, a critical component in NFκB activation.

Hepatotropic(+) RNA viruses are sensed by key specific pattern recognition receptors (PRRs) that are abundantly expressed in the liver^31^, such as RIG-I-like receptors (RLRs) and Toll-like receptors (TLRs). These evolutionarily conserved receptors typically sense dsRNA produced during viral replication. Cytoplasmic RLRs include retinoic acid-inducible gene I (RIG-I), melanoma differentiation-associated gene 5 (MDA5) and laboratory of genetics and physiology 2 (LGP2). RIG-I recognizes short dsRNA (<500 base pairs) with 5′ triphosphorylated ends, while MDA5 detects longer dsRNA. Upon binding dsRNA, MDA5 and RIG-I undergo conformational changes that expose their N-terminal caspase activation and recruitment domains (CARD). The exposed CARD domains interact with the mitochondrial antiviral signaling (MAVS) adapter molecule, which prompts TANK-binding kinase 1 (TBK1) and IκB kinase-ε (IKKε) to phosphorylate transcription factors, including IFN regulatory factor 3 (IRF3), IRF7 and NFκB. These activated factors form dimers that migrate to the nucleus, promoting the transcription of genes encoding IFN, IFN-stimulated genes (ISGs) and immunoregulatory genes (reviewed in ref. ^32^) (Fig. 2).

In contrast with RIG-I and MDA5, LGP2 recognizes various RNA molecules regardless of length or 5′ phosphate ends, and lacks the CARD domain. LGP2 contributes to innate immune signaling by enhancing the recognition of dsRNA by MDA5 and by inhibiting RIG-I^33^.

Primary human hepatocytes derived from chimeric mice with humanized livers (PhoenixBio, PXB-mice) and fetal rhesus kidney cells (FRhK4) are immune-competent cells that express physiological levels of PRRs and were not initially investigated for their innate immune responses to HAV^34,35^. In contrast, clones derived from the hepatoma cell line Huh7 show impaired PRR expression and were found to be highly permissive toward HAV^25^. The HAV genome, with its covalently linked 5′ VPg, is unlikely to be recognized by RIG-I, which identifies RNAs with free 5′ triphosphate ends. On the other hand, MDA5 reportedly senses multiple picornaviruses, including HAV. Sung et al.^36^ confirmed this finding in immune-competent models, including primary human hepatocytes (PHH) and immortalized HepG2 cells. In these models, induction of the chemokine CXCL10 was strictly dependent on MDA5-based sensing of HAV^36^. Another study verified the role of MDA5 as the main HAV sensor in infected HepaRG cells, demonstrating the detection of ISGs and CXCL10 upon HAV infection, and also reported the pivotal contribution of LGP2 to HAV sensing^25^.

With regards to TLR3, Qu et al. reported that Huh7 cells ectopically expressing TLR3 did not exhibit IFN induction upon HAV infection, suggesting viral counteraction toward the adapter protein TRIF, rather than a lack of induction^17^. Successful TLR3 sensing would rely on HAV dsRNA reaching the endosomal lumen, but this has not yet been experimentally tested. Importantly, the literature also shows that plasmacytoid dendritic cells (pDCs) can identify eHAV, thereby mounting an innate immune response based on IFN-α secretion and robust ISG induction in vitro^37^. However, in HAV-infected chimpanzees, pDCs were only transiently observed and ISGs were not elicited upon HAV infection, even though the infected chimpanzees exhibited strong viremia^22^. Again, this finding appeared linked to an ability of HAV to interfere with the innate immune response through various proteins, which will be examined in the next section.

## HAV-mediated counteraction against innate immunity

**Fig. 3:**
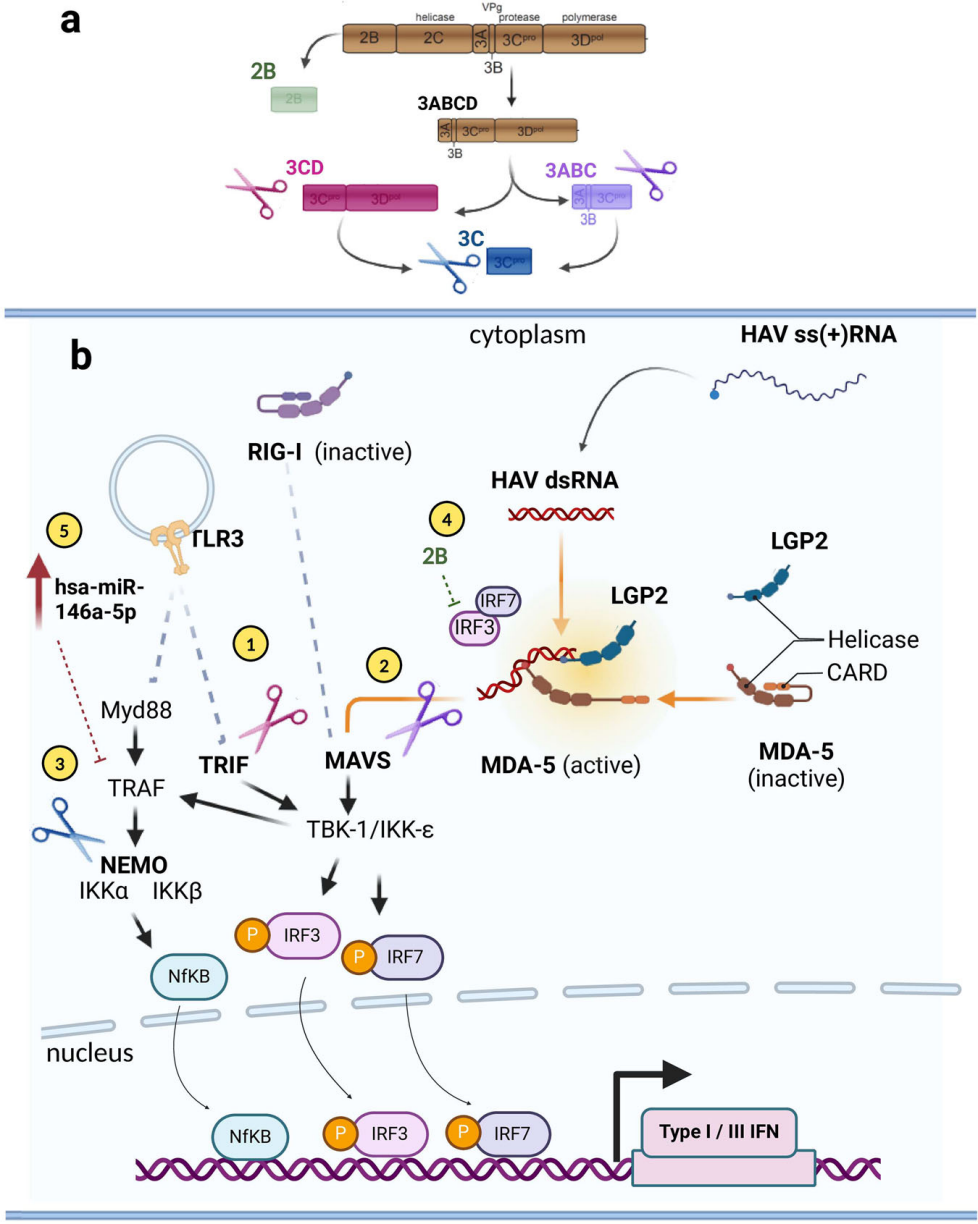
Mechanisms by which HAV counteracts innate immunity. A schematic representation of the strategies by which HAV reportedly interferes with innate immunity. **a** The cysteine protease HAV 3C^pro^ undergoes sequential autoprocessing of the P3 polyprotein precursor—involving sequential liberation into single intermediates, starting with 3ABCD, followed by 3ABC and 3CD and finally 3C. The protein 2B is also depicted for its role in counteracting innate immunity. **b** The 3CD reportedly cleaves TRIF, the adapter protein mediating TLR3 signaling (1). The 3ABCpro intermediate cleaves MAVS (2). NEMO is a target of direct cleavage by 3Cpro (3). The nonstructural HAV protein 2B is believed to interact with MAVS, disrupting the activity of TBK1 and IKKε kinases, although the precise mechanism is unclear (4). Recent studies show that HAV infection increases the expression of microRNA hsa-miR-146a-5p, which targets and degrades TRAF6 mRNA (5).

The cysteine protease HAV 3C^pro^ undergoes hierarchical autoprocessing of the P3 polyprotein precursor—involving sequential liberation into single intermediates, starting with 3ABCD, followed by 3ABC and 3CD and finally 3C (Fig. 3a). These 3C species possess varying stability, with processing at the 3CD site reported to be more efficient compared with at 3AB and 3BC^38^. All species exhibit retained proteolytic activity and distinct substrate specificities, and all are potentially capable of cleaving a variety of host molecules involved in innate immune signaling.

Yang et al. first reported that 3ABC^pro^ and 3ABCD^pro^ can proteolytically cleave MAVS, in a study that demonstrated MAVS degradation in both HAV-infected cells and cells harboring a HAV subgenomic replicon^23^. They further showed that 3ABC cleavage of MAVS requires the protease activity of 3C^pro^ and a transmembrane domain in 3A that directs 3ABC to the mitochondria. Moreover, the authors demonstrated a corresponding hindrance of RLRs-mediated pathways through an experiment using a luciferase reporter under the control of an IFN-β promoter, in the presence of an external immunostimulant. Qu et al. later employed similar models with addition of a cell-free cleavage assay, which demonstrated the robust proteolytical degradation of TRIF by 3CD, but not by the mature 3C^pro^ or the 3ABC precursors^17^. Notably, the TRIF molecule does not contain canonical 3C^pro^ consensus cleavage sites: ((L,V,I)X(S,T)Q↓X, in which X is any amino acid and ↓ indicates the protease cleavage site. Rather, TRIF contains sites that are only partial fits. Qu et al.^17^ demonstrated that the 3CD-mediated cleavage of TRIF depends on both the cysteine protease activity of 3C^pro^ and the downstream 3D^pol^ sequence, independent of 3D^pol^ activity. They suggested that the 3D polymerase might promote ‘tolerance’ for acidic residues at the P4 position of TRIF cleavage sites, enabling scission by 3C + 3D (3CD), but not by 3C alone. However, currently available experimental evidence does not elucidate the mechanism by which 3D redirects the cleavage specificity. Nonetheless, Qu et al.^17^ showed that TRIF abrogation led to functional ablation of the TLR3 pathway. These results were later verified by Fensterl et al.^20^, who proposed that HAV may interfere with the phosphorylation of TBK1 and IKKε, thereby reducing the efficiency of TRIF downstream signaling.

A later study confirmed the proteolytic^25^ cleavage of MAVS by 3ABC and TRIF by 3CD, although with lower efficiency than previously reported. Importantly, no abrogation of innate immune functions was detected using approaches involving HAV subgenomic replicon cell lines, HAV-infected cells and cells stably expressing the distinct HAV protease species. In these cells, the TLR3 and RLR pathways were intact and responsive to treatments with immunostimulants, despite robust expression of the HAV protease precursors^25^.

Evolving views continue to appear in the literature as the body of evidence grows. An important study by Hirai-Yuki demonstrates that MAVS knock-out (KO) in wild-type mice increases susceptibility to HAV infection, suggesting that RLR pathway evasion is a pivotal strategy evolved by HAV (and other viruses) to survive the innate immune response and the inflammation mediated by the RLR–IRF3 axis^39^. However, Sun et al.^40^ recently showed that when MAVS KO mice were reconstituted with a ‘humanized MAVS’ molecule including specific cleavage sites for HAV 3ABC, they did not exhibit increased susceptibility to HAV infection. This important piece of in vivo evidence indicates that MAVS cleavage may not be as critical for successful HAV infection as previously suggested. Additionally, Hirai-Yuki reported that TRIF KO mice, with full ablation of the TLR3 pathway, did not exhibit increased permissiveness towards HAV infection^39^.

In another study, Wang et al. demonstrated that NEMO undergoes direct cleavage by 3C^pro^, leading to TLR3–IRF3 pathway disruption and downregulation of IFN-β transcription^18^. Further investigations are required to elucidate the functional impact of NEMO cleavage.

Apart from protease-dependent mechanisms, HAV reportedly employs additional strategies to abrogate the host immune response. The nonstructural HAV 2B protein was shown to be capable of disrupting TBK1/IKKε kinase activity. Paulmann et al. suggested that both TBK1 and MAVS may be affected by a concerted action of HAV proteins 2B and 3ABC; however, the exact mechanism remains to be clarified^19^. Furthermore, it was recently reported that HAV infection increases the expression of microRNA hsa-miR-146a-5p, which targets and degrades TRAF6 mRNA, affecting IFN-β synthesis and increasing viral replication^24^.

Overall, HAV is apparently capable of counteracting innate immunity through multiple mechanisms; however, none seems to fully abrogate the pathways, and thus IFN and ISG responses still occur upon HAV infection. Figure 3b shows an overview of the currently proposed mechanisms of innate immune interference by HAV.

## HAV-induced innate immune responses in in vivo models

**Fig. 4:**
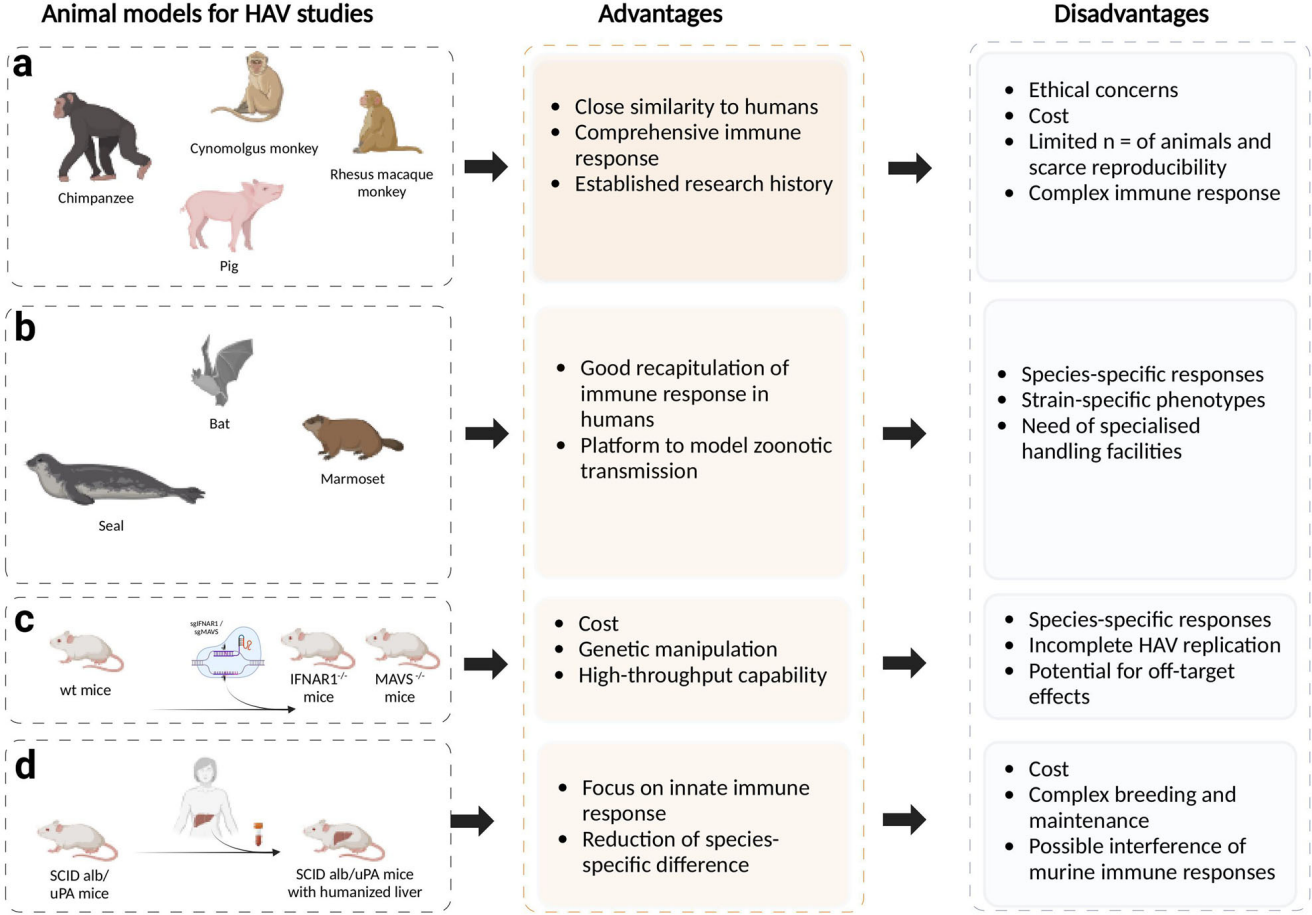
HAV in vivo infection models currently available to assess innate immunity. A schematic of different animals that are supportive of HAV infection, showing the advantages and disadvantages of their utilization. **a** Primates and pigs. **b** Mammals prone to infection with HAV-like viral strains. **c** Wild-type (WT) mice with KO of MAVS or IFNAR1. **d** Albumin-urokinase-type plasminogen activator/severe combined immunodeficient (Alb/uPASCID) mice with humanized livers.

HAV infection has been explored in various animal models in the context of innate immune studies. Primates and non-human primates—such as chimpanzees, marmosets and owl monkeys—offer valuable insights due to their similarities to humans. However, their use is constrained by limited availability and ethical issues^41^ (Fig. 4a). When pigs were successfully infected with human HAV strains, inflammatory cell infiltration was observed in the livers of infected animals but not in controls, and the immune response generally resembled that observed in humans and primates^42^. Furthermore, recent findings of various HAV strains in animals—including seals, bats, rodents, hedgehogs, ducks and woodchucks—may broaden the spectrum of animal models available for HAV research^41^ (Fig. 4b).

Among the small animals available for HAV immune studies, wild-type mice are not permissive toward HAV without KO of MAVS or IFNAR1. This underpins the relevance and strength of an HAV-elicited immune response, which directly correlates to viral clearance in humans^39^ (Fig. 4c). Experiments in animals that show functional adaptive immune responses for infections that are robustly cleared, such as HAV, leave many unanswered questions about the role of the innate immune response. Specifically, in MAVS/IFNAR1 KO mice, HAV infections reportedly acquire persistence, highlighting gaps in our understanding of the innate immune response. In this regard, the development of chimeric mice with humanized livers has been essential^43^. These mice express a combination of harmful genes under the control of an albumin promoter, leading to the degradation and necrosis of murine hepatocytes, allowing repopulation with PHH. Human liver chimeric mice lack the main professional adaptive immune cells; this supports infections with human hepatotropic viruses, thus providing a valuable model (Fig. 4d). A recent study used this model to examine the innate immune response upon HAV infection^25^. Here, expression of the 3C viral antigen was correlated with elicitation of type-III IFN, multiple ISGs and chemokines in the infected human hepatocytes, clarifying the successful sensing of HAV dsRNA in infected cells and arguing against HAV showing a strong and ablating counteraction strategy. This HAV-triggered cell-intrinsic innate immune response might lead to recruitment of professional immune cells and ultimately contribute to the establishment of clearance.

## Virus-specific T cell responses in HAV infection

Early studies explored the role of HAV-specific CD8^+^ T cells in AHA. Vallbracht et al.^44,45^ described IFN-γ-releasing lymphocytes in the blood and liver of patients with HAV infection, which could lyse HLA-class I-matched HAV-infected fibroblasts. Notably, these lymphocytes did not lyse HLA-class I-mismatched fibroblasts, or matched fibroblasts that were uninfected or infected with other viruses^44,45^. Since those reports, advanced methods have been developed for studying antigen-specific T cells^46,47^, enabling more recent studies to directly characterize HAV-specific T cells ex vivo.

Schulte et al.^48^ were the first to report HAV-specific CD8^+^ T cell epitopes. They utilized an epitope prediction tool to identify epitopes that bind to HLA-A2, and used epitope peptides to stimulate PBMCs from patients with HAV infection. After finding one dominant peptide in the 3D protein of HAV, which was highly conserved across different HAV genotypes, they used it to synthesize an HLA-A2 tetramer. They next used this tetramer to characterize HAV-specific CD8^+^ T cells in patients with HAV infection as being activated during early infection and as exhibiting a memory phenotype in the postinfection stage^48^.

In a chimpanzee model, Zhou et al.^49^ conducted an in-depth investigation of the characteristic changes of HAV-specific CD8^+^ and CD4^+^ T cells relative to viral titer and liver enzyme variations. They found that the proportion of HAV-specific tetramer^+^ CD8^+^ T cells was highest after the viral titer and hepatitis had decreased. Notably, at this time point, only about 20% of the cells secreted IFN-γ or TNF upon stimulation with MHC class I epitopes. When the viral titer persisted, the frequency of tetramer^+^ CD8^+^ T cells sharply decreased. In contrast, HAV-specific CD4^+^ T cells—which were identified by stimulation with pooled MHC class II epitopes—secreted multiple cytokines as the viral titer began to decline. The proportion of functional CD4^+^ T cells increased with transient fecal RNA resurgence and was gradually reduced with decreasing viral load^49^. These findings suggested that CD4^+^ T cells may directly or indirectly play an important role in viral clearance.

**Fig. 5:**
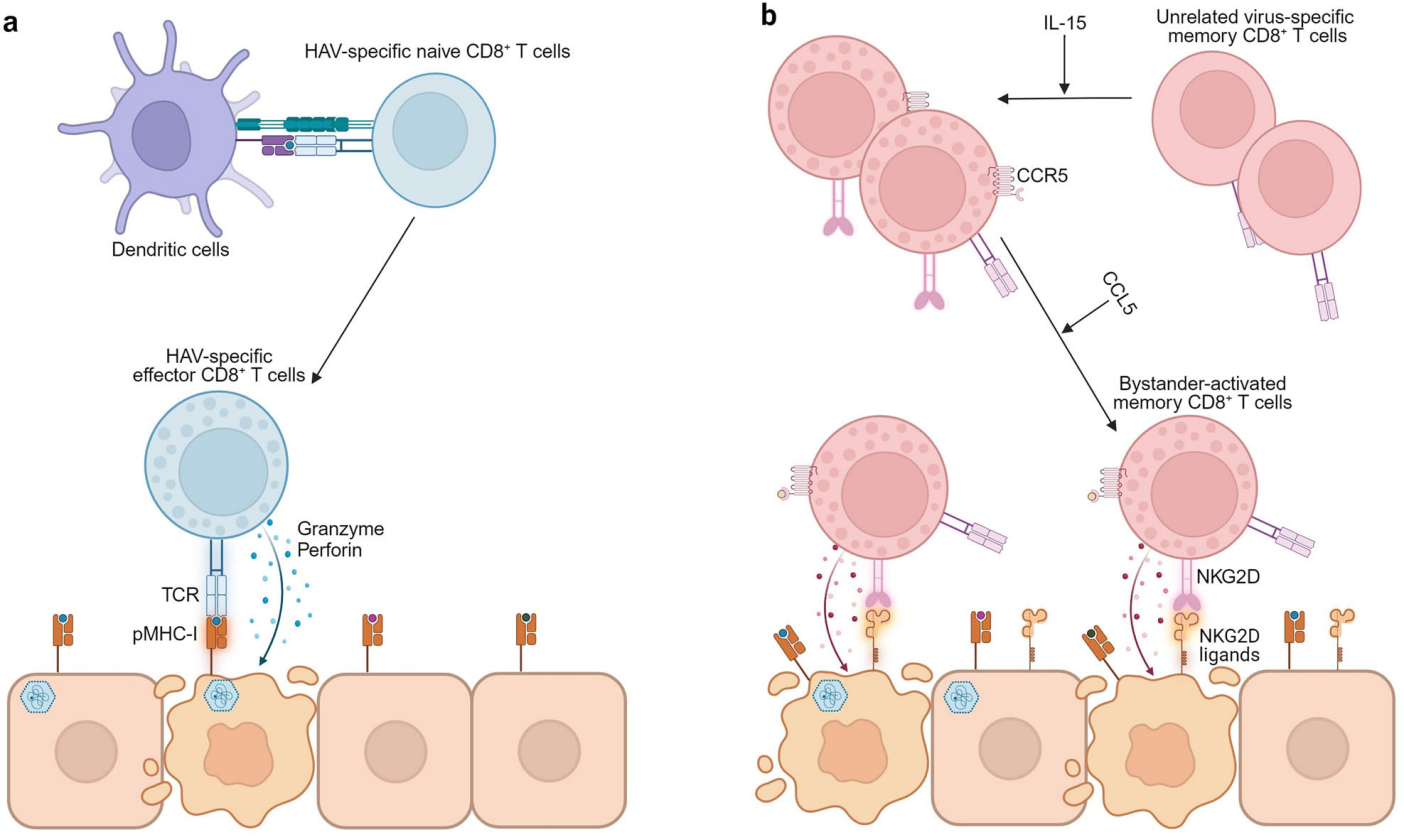
Roles of CD8^+^ T cells in HAV infection. **a** During HAV infection, naive CD8^+^ T cells specific to HAV are activated by dendritic cells in the lymph nodes. Subsequently, HAV-specific effector CD8^+^ T cells migrate to the liver and selectively eliminate HAV-infected cells via a TCR-dependent mechanism. **b** During HAV infection, elevated IL-15 activates pre-existing memory CD8^+^ T cells specific to unrelated viruses. These IL-15-activated bystander memory CD8^+^ T cells show increased expressions of NKG2D and CCR5. The bystander-activated memory CD8^+^ T cells migrate using the CCR5–CCL5 axis and exert NK-like cytotoxicity, targeting hepatocytes expressing NKG2D ligands to be killed. The innate-like cytotoxicity of bystander-activated CD8^+^ T cells is correlated with serum ALT levels, indicating liver damage in AHA.

Misumi et al. used an infection model of *Ifnar1*^*−*/−^ mice, and revealed that CD8^+^ T cells play an important role in HAV clearance^39^. HAV-specific CD8^+^ T cells, defined using tetramers, exhibited an inverse correlation with HAV viral titers and alanine aminotransaminase (ALT) levels. This correlation was not observed with HAV-specific CD4^+^ T cells, which were also defined with tetramers. In T cell depletion experiments, CD4^+^ and CD8^+^ T cell depletion each resulted in increased viral titers and worsened hepatitis. However, when these populations were stimulated using a peptide vaccine, only the increase of CD8^+^ T cells resulted in improvement. There remains a need for further research using immunocompetent models to determine which population of HAV-specific T cells is crucial for HAV clearance (Fig. 5a).

## Virus-nonspecific IL-15-induced activation of T cells in HAV infection

AHA is mostly a self-limiting disease, but it can sometimes lead to fulminant hepatic failure, necessitating liver transplantation. Severe liver injury is more commonly observed in primary infection of adults, compared with children^50^. Since HAV is noncytopathic, it has been hypothesized that liver damage is caused by the host’s immunity^15,51,52^. However, it has long been unclear how the immune response leads to liver damage in adults with AHA.

A recent series of studies suggested a possible answer to this question^26,53^. HLA-class I tetramers were used to analyze CD8^+^ cells from patients with AHA, revealing the activation of both HAV-specific CD8^+^ T cells and HAV-unrelated virus-specific CD8^+^ T cells. Notably, IL-15—which increases in the liver and serum during HAV infection—was identified as a crucial cytokine for the TCR-independent bystander activation of HAV-unrelated virus-specific memory CD8^+^ T cells^26^. Furthermore, bystander CD8^+^ T cells that were activated via an IL-15-induced TCR-independent mechanism exhibited increased expression of NKG2D, NKp30 and CCR5 (refs. ^26,53^). Hepatocytes in HAV-infected livers exhibited increased levels of the NKG2D ligand MICA/B. In vitro experiments demonstrated that bystander-activated CD8^+^ T cells could migrate using the CCR5–CCL5 axis and kill target cells via the NKG2D and NKp30 pathways. In patients with AHA, the activation of HAV-unrelated virus-specific bystander CD8^+^ T cells was strongly correlated with serum ALT levels, but not with viral titer. In contrast, HAV-specific CD8^+^ T cell responses were inversely correlated with viral titer and serum ALT levels. Taken together, these findings indicate that during acute HAV infection, bystander-activated CD8^+^ T cells play a crucial role in liver injury but not in viral control, and that HAV-specific CD8^+^ T cells contribute to both virus elimination and the control of liver injury (Fig. 5a, b)^26^. Recent studies include reports that CD8^+^ T cells activated in an IL-15-induced TCR-independent manner may also contribute to liver or endothelial cell injury in other viral infections^54,55^. These findings indicate that despite other possible causes of liver damage^39^, the predominance of severe hepatitis in adults with AHA can probably be attributed to the higher number of pre-existing memory CD8^+^ T cells, which can be activated through an IL-15-induced mechanism in adults compared with children. On the other hand, excessive IL-18 responses caused by a deficiency of IL-18-binding protein also contribute to liver damage through natural killer (NK) cell activation^56^.

Mucosal-associated invariant T (MAIT) cells have also been studied in the context of IL-15-induced TCR-independent activation. Rha et al.^57^ demonstrated that IL-15 also activates liver MAIT cells, which comprise the majority of innate-like T cells in the liver^58^. IL-15-activated liver MAIT cells killed target cells via NKG2D dependency and CD2 conjugation without TCR/MR1 engagement. This innate-like cytotoxicity of liver MAIT cells is regulated via the PI3K–mTOR pathway, and has been associated with hepatic injury in patients with AHA.

## Dysregulation of the Treg cell population in HAV infection

Treg cells play a role in suppressing cellular immunity to ameliorate tissue damage and interfere with viral clearance in viral hepatitis^59–61^. However, in AHA, Treg cells exhibit decreased frequency and functional dysregulation.

**Fig. 6:**
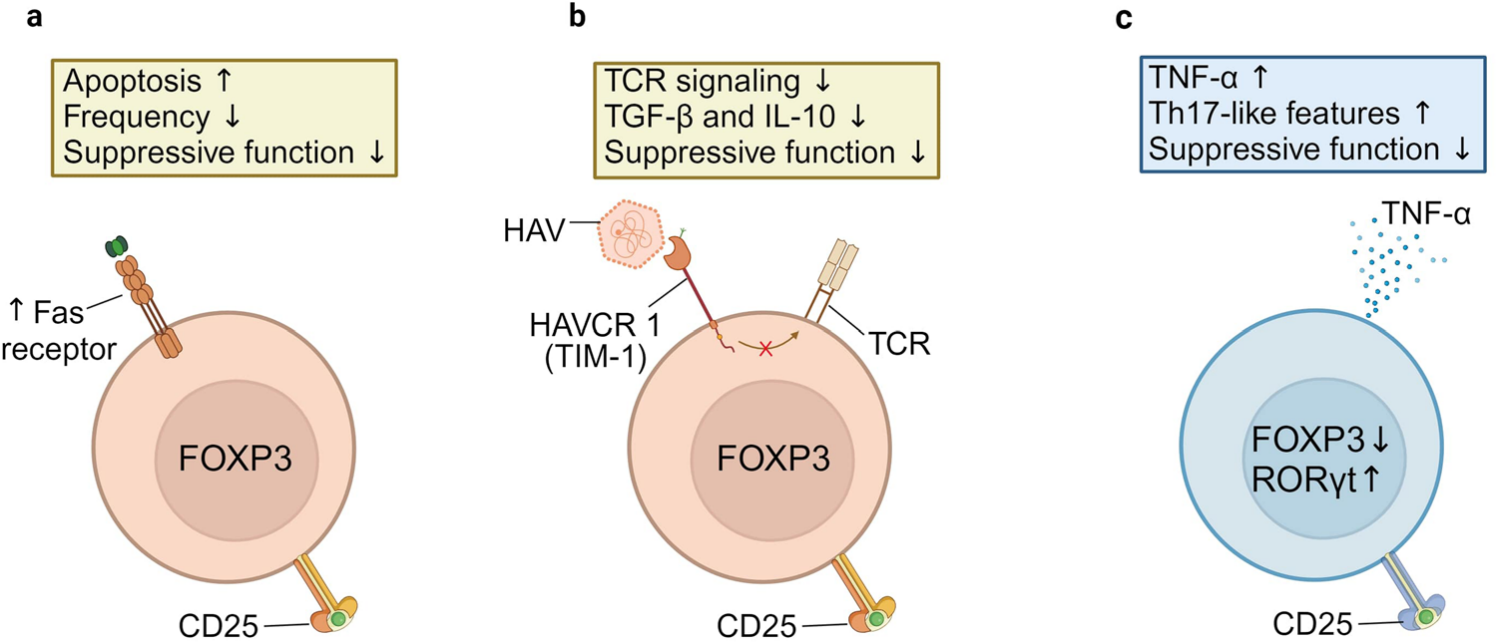
Quantitative and qualitative changes in the Treg cell population in HAV infection. **a** During HAV infection, the frequency of Treg cells is decreased through Fas-mediated apoptosis, leading to reduced suppressive function of the Treg cell population. **b** The binding of HAV to HAVCR1 prevents TCR activation, resulting in reduced release of suppressive cytokines by Treg cells. In AHA, the frequency and suppressive function of the Treg cell population are inversely correlated with liver damage. **c** During AHA, there is an increased frequency of Th17-like Treg cells that produce TNF through an RORγt-dependent mechanism. The frequency of TNF-producing Treg cells is correlated with severe liver damage in AHA.

Choi et al.^62^ reported that the Treg cell population from patients with AHA exhibited decreased frequency and reduced suppressive function, compared with healthy controls. Notably, Treg cells from patients with AHA showed increased Fas expression and susceptibility to Fas-mediated apoptosis. This suggests that in patients with AHA, Treg cell apoptosis occurs through the Fas–Fas ligand pathway. The increased Fas expression on Treg cells was a unique feature of AHA, and was not observed in Treg cells from patients with other forms of hepatitis^62^ (Fig. 6a).

In patients with AHA, Treg cells are also functionally changed. One study highlighted the importance of HAV cellular receptor 1 (HAVCR1) in regulating the suppressive function of Treg cells^63^. HAVCR1—also known as T cell immunoglobulin and mucin domain-containing protein 1 (TIM-1)—is a receptor for phosphatidylserine expressed on apoptotic cells and for cellular entry of HAV^64,65^. HAVCR1 expressed on Treg cells binds to HAV, inhibiting TCR activation and leading to decreased secretion of suppressive cytokines and reduced suppressive function of Treg cells^63^ (Fig. 6b).

Choi et al.^27^ reported that compared with Treg cells from healthy controls, Treg cells from patients with AHA exhibited both decreased suppressive function and increased proinflammatory function. In Treg cells from patients with AHA, ex vivo TCR stimulation elicited the production of TNF, as well as IFN-γ and IL-17A. These cells also exhibited increased RORγt and CCR6 expressions, and RORγt expression was required for TCR-induced TNF production. Notably, DNA methylation analysis confirmed that TNF-producing Treg cells were bona fide Treg cells (Fig. 6c). It was recently reported that in vitro TNF stimulation increases the TNF-producing capacity of Treg cells obtained from healthy donors^66^. These findings suggest that the TNF-producing capacity of Treg cells is enhanced by abundant TNF secreted by several types of inflammatory immune cells during AHA.

In HAV infection, Treg cell frequency changes and dysfunction have been linked to liver damage. In patients with AHA, ALT levels were found to be inversely correlated with Treg cell frequency and suppressive function, but positively correlated with the proportion of TNF-producing Treg cells^27,63^. Additionally, an HAVCR1 (TIM-1) polymorphism that enhances binding to HAV is reportedly associated with HAV infection-induced severe liver disease^67^. Overall, the presently available evidence is indirect and further direct studies are needed to establish a definitive link between Treg cells and HAV infection-induced liver damage.

## Conclusions

In this review, we summarize recent findings on the immune response in HAV infection, including cell-intrinsic innate immune responses in HAV-infected hepatocytes and IL-15-induced activation of bystander CD8^+^ T cells in patients with HAV infection. Despite the development of effective vaccines against HAV infection, AHA is the most common type of acute viral hepatitis. In HAV infection, immune responses can contribute to both viral clearance and liver damage. Further studies are needed to address key questions on how immune responses are regulated in the context of the balance between beneficial and detrimental immune responses during HAV infection. Better understanding of the immune response and immunopathogenesis of HAV infection will also provide knowledge for better management of other human viral diseases.
